# Insufficient transparency of statistical reporting in preclinical research: a scoping review

**DOI:** 10.1038/s41598-021-83006-5

**Published:** 2021-02-08

**Authors:** Romain-Daniel Gosselin

**Affiliations:** Precision Medicine Unit, Lausanne University Hospital, Chemin des Roches 1a/1b, 1010 Lausanne, Switzerland

**Keywords:** Data publication and archiving, Experimental organisms, Animal disease models

## Abstract

Non-transparent statistical reporting contributes to the reproducibility crisis in life sciences, despite guidelines and educational articles regularly published. Envisioning more effective measures for ensuring transparency requires the detailed monitoring of incomplete reporting in the literature. In this study, a systematic approach was used to sample 16 periodicals from the ISI Journal Citation Report database and to collect 233 preclinical articles (including both in vitro and animal research) from online journal content published in 2019. Statistical items related to the use of location tests were quantified. Results revealed that a large proportion of articles insufficiently describe tests (median 44.8%, IQR [33.3–62.5%], k = 16 journals), software (31%, IQR [22.3–39.6%]) or sample sizes (44.2%, IQR [35.7–55.4%]). The results further point at contradictory information as a component of poor reporting (18.3%, IQR [6.79–26.7%]). No detectable correlation was found between journal impact factor and the quality of statistical reporting of any studied item. The under-representation of open-source software (4.50% of articles) suggests that the provision of code should remain restricted to articles that use such packages. Since mounting evidence indicates that transparency is key for reproducible science, this work highlights the need for a more rigorous enforcement of existing guidelines.

## Introduction

Reliable biomedical research is intertwined with sound experimental design, adequate statistical analysis and fully transparent communication of protocols and results to ensure adequate third-party data interpretation, the replication of studies and the capacity to perform meta-analyses. Despite this, insufficiently reported statistics are widespread, contributing to the so-called reproducibility crisis^1–3^. This situation is particularly disturbing in preclinical science where the mishandling of statistics may both lead to the unethical use of large numbers of laboratory animals and complicate subsequent clinical investigations by increasing the number of clinical studies that are unnecessary or potentially harmful for patients^4^. Scores of guidelines exist, many of which being compiled on the Enhancing the QUAlity and Transparency Of health Research (EQUATOR, https://www.equator-network.org/) network. The leading guidelines used in preclinical research is the Animal Research: Reporting of In Vivo Experiments (ARRIVE), which has been recently updated (ARRIVE 2.0 version)^5, 6^ but other official guidelines exist such as the one published by the American Physiological Society^7^, the Checklist for Reporting In-vitro Studies (CRIS) guidelines^8^ and the checklist by Emmerich and Harris for in vitro research^9^. However, series of scoping reviews have documented that unclear and non-transparent reporting of statistical methods remain in the life preclinical literature^2, 10–13^, prompting the conclusion that these guidelines have had limited impact thus far^14–16^. Therefore, more coercive enforcement of rigorous reporting standards, existing or yet to come, by scholarly editors might be necessary. Notably, the aforementioned scoping reviews examined the literature mostly in animal research, although a lack of transparency in preclinical science involving other approaches also constitute a threat to reproducibility. The rigorous documentation of the most frequent reporting practices and mistakes in the broad spectrum of preclinical research would be necessary to refine the existing guidelines, tailor new policies and build innovative educational programmes.

The present scoping review aims at providing a recent quantification of insufficient reporting of statistical methods in a large sample of preclinical publications, from in vitro to animal research. The results indicate that under-reporting is ubiquitous and that even the most elementary statistical information is not consistently presented transparently.

## Results

**Figure 1:**
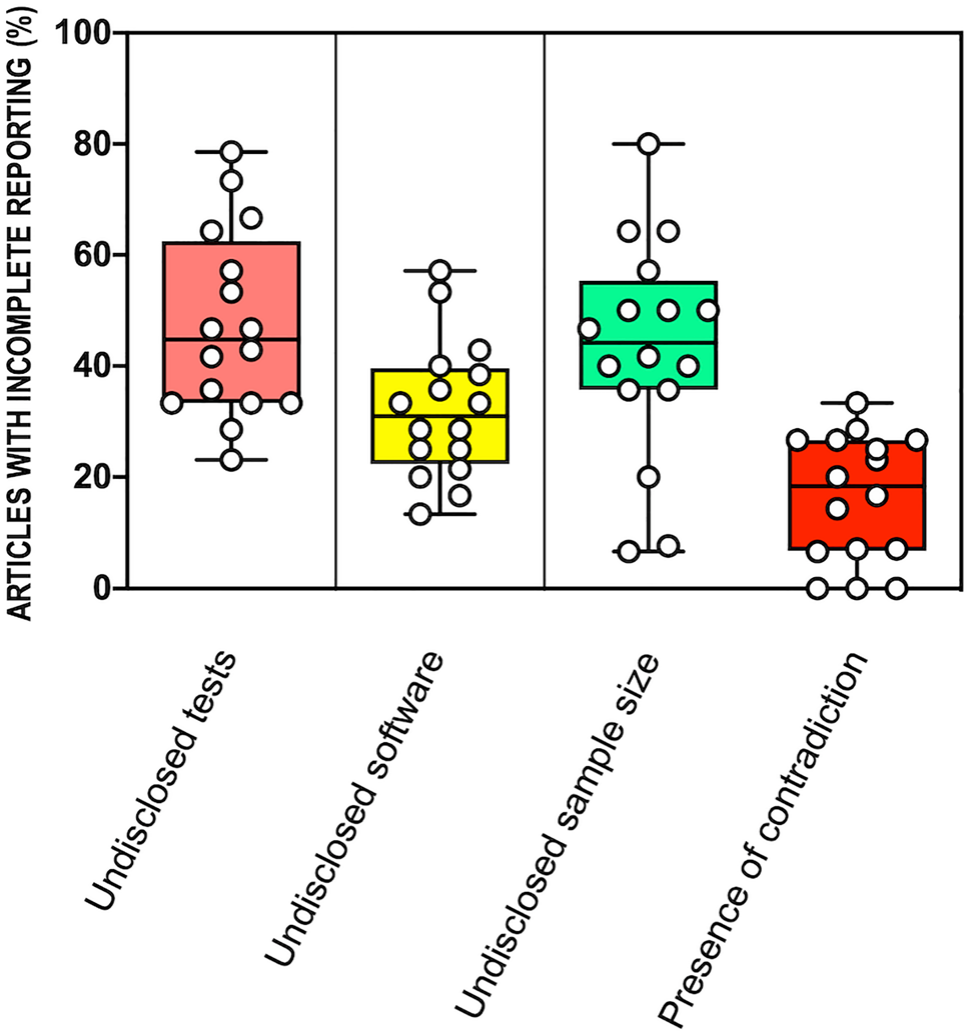
Quantification of primary binary outcomes. The Y axis displays the percentage of articles with incomplete reporting in each journal, each dot represents one journal (k = 16). Box plots represent interquartile range (box) and range (whiskers).

**Figure 2:**
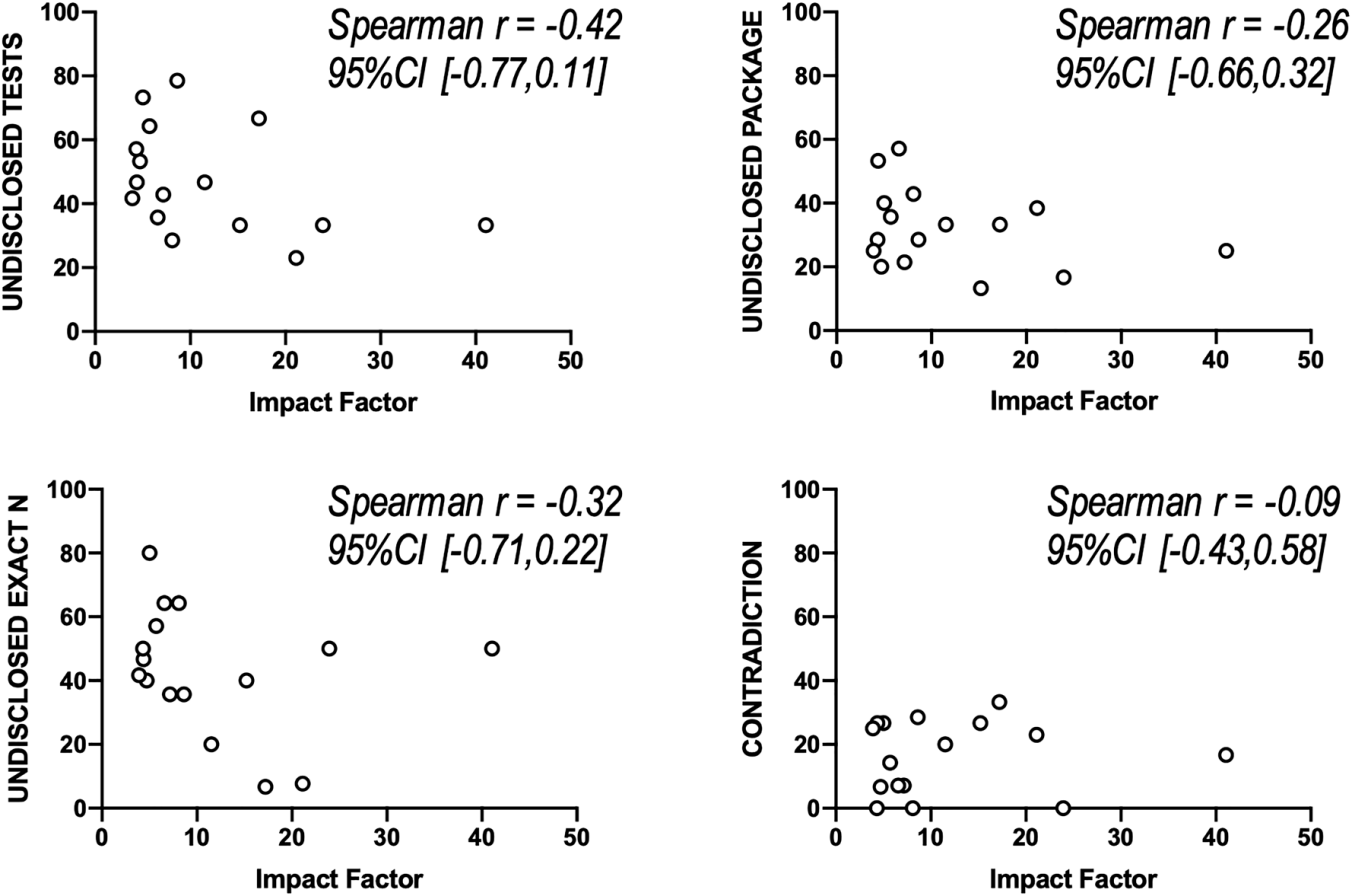
Scatter plots showing the correlation study between percentage of incomplete reporting and journal impact factor (2018). 95% confidence interval of Spearman correlation coefficient are given between brackets. Each dot represents one journal (k = 16).

The descriptive analysis of quantitative outcomes in the sample of journals revealed a median number of figure or tables per article of 6.66 (range [4.58–9.08], k = 16 journals) and among these, a large proportion display results of at least one location test (i.e. that allow to test hypotheses about population means or medians; median 79.72%, range [43.22–95.41%], k = 16). In addition, the absence of a dedicated paragraph describing statistical methods was an infrequent albeit not exceptional chosen presentation (median 3.34%, range [0–33.33%], k = 16). Figure 1 shows the quantification of binary outcomes (i.e. related to the quality of reporting in figures and tables). Insufficient disclosure of tests (median 44.8%, interquartile range (IQR) [33.3–62.5%], k = 16 journals), packages (median 31%, IQR [22.3–39.6%], k = 16) and exact sample sizes (median 44.2%, IQR [35.7–55.4%]), k = 16) occurred particularly frequently. A notable proportion of articles (median 18.3%, IQR [6.79–26.7%]), k = 16) present contradictory information. A contradiction is defined as a mismatch between information provided in different parts of the manuscript although they refer to the same object, such as the disclosure of dissimilar statistical tests (in methods and figure legends) to describe the analysis in one figure or the disclosure of multiple sample sizes for one single set of data. The possible relationship between journal impact factor and the frequency of incomplete reporting in articles was explored (Fig. 2) and no statistically significant correlation could be detected either for test disclosure (Spearman r = -0.42, 95% confidence interval (CI) [-0.77–0.11], p = 0.1042, k = 16 journals), package disclosure (Spearman r = -0.23, 95% CI [-0.66–0.32], p = 0.3982, k = 16), sample size disclosure (Spearman r = -0.32, 95% CI [-0.71–0.22], p = 0.2221, k = 16) or presence of contradiction (Spearman r = 0.1, 95% CI [-0.43–0.58], p = 0.7216, k = 16).

**Figure 3:**
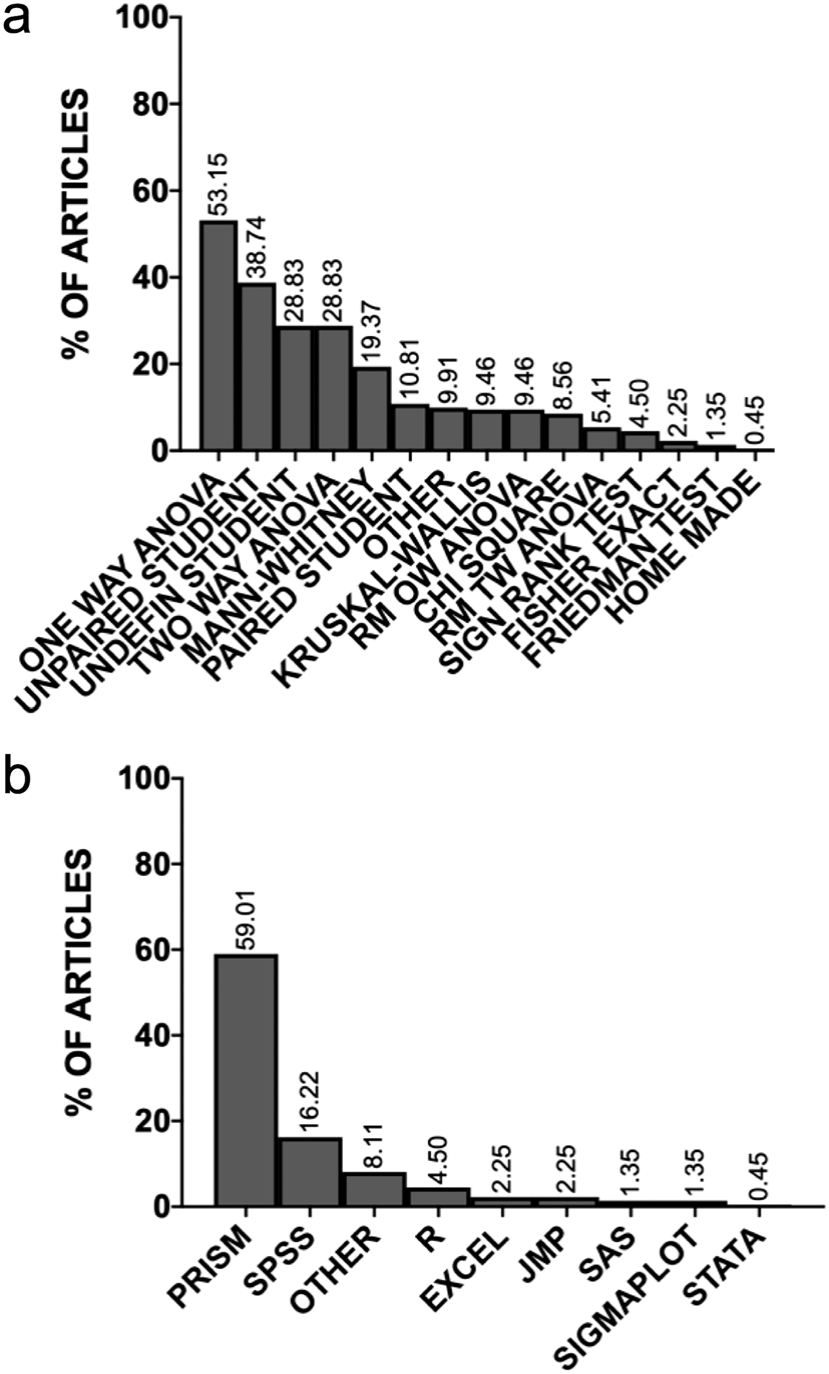
Histograms showing the frequency distributions of tests (A) and software (B). The Y axis gives the percentage of articles with the given item. Note that the overall sum of percentages may be above 100% since more than one test (in A) or type of software (in B) may be present in a given article (k = 223 articles).

The analysis of frequency distribution of location tests used in articles is presented in Fig. 3A. The most frequently used tests were one way analysis of variance (ANOVA; used in 53.15% of articles, k = 223 articles), two way ANOVA (28.83%), repeated measure one way ANOVA (9.46%), unpaired Student’s t test (38.74%) and Student’s t test of undefined laterality (26.83% of articles). Non-parametric tests were less frequently used than their parametric counterparts. Of these, the Mann–Whitney test (19.37% of articles) was the most frequently applied. Finally, the frequency distribution of statistical packages used in articles is presented in Fig. 3B. The most frequently used software was determined to be Prism (mentioned in 59.01% of publications, k = 223) and SPSS (16.22%). The only non-proprietary package mentioned in the sampled articles is R (used in 4.50% of articles).

## Discussion

The validity of preclinical publications is called into question due to the tolerance to unsound statistics, including a lack of transparency in reporting^12, 17^. In an attempt to isolate the shortcomings that persist in spite of existing guidelines, the most frequent statistical features and indicators of non-transparent reporting were systematically documented in 223 articles published in 2019 in 16 journals. The results are confirmatory of the current representation of transparency in life science by pointing at insufficient reporting of tests, sample size and software. The study also updates this knowledge by identifying contradictory information as a contributor to poor reporting and by suggesting that preclinical guidelines should probably not immediately insist on the comprehensive provision of the code run for data analysis unless an open-source software was used.

Results showed that location tests are highly prevalent but are reported using insufficient standards in preclinical literature, both of which justify the orientation of the present study. In accordance with previous reports^18^, the results point to the entrenched culture of using parametric tests in life sciences and therefore emphasises the importance of educating researchers regarding the specificities of reporting information on parametric testing (e.g. whether parametric assumptions were verified and how). The deficiencies in reporting most frequently identified pertain to test, sample size and package disclosure, all reaching alarming proportions. This is in accordance with previous studies that pointed at a marked proportion of animal research with insufficient information about sample size and statistical procedures^11, 13^. Interestingly, journal impact factor did not seem to be statistically correlated with the number of insufficiencies identified in articles. Previous reports by others also indicated inconsistent correlation between journal impact factor and the quality of reporting^10, 13, 19, 20^.

Finally, the omnipresence of proprietary software such as GraphPad Prism, which are based on a graphical user interface (GUI) and whose codes are generally not accessible (unlike open-source packages), strongly suggests that the mandatory disclosure of code may be very difficult at the moment in preclinical science. This situation is different from what has been recommended in other fields^21^. Therefore, the full disclosure of the software used (including the exact version and complete description of commands implemented) might remain acceptable at present in articles using packages such as Prism. However, it should become mandatory that such companies make publishable scripts more easily accessible to users. In a near future, more journals in preclinical science should ultimately request the code or script of analysis of GUI packages, even though authors did not create the command-lines themselves.

The real impact of the numerous existing guidelines has been limited^13–16^ although many of the pinpointed shortcomings could be efficiently corrected at no significant extra cost by the adoption of simple measures^22^. Various strategies may be envisioned to improve reporting, such as a more coercive enforcement of existing guidelines by scholarly publishers, an increased awareness of their existence or the creation of unified guidelines aiming to reduce their multiplicity. It is crucial that both the editorial system, research institutions and coordinators of graduate programmes take seriously the importance of the statistical training of current and future peer-reviewers, in particular with respect to reporting. Journals might also systematically recruit statistical reviewers or peer-reviewers with a marked literacy in statistical reporting. In addition, educational programmes in design and applied statistics for graduate students and researchers that are currently blossoming worldwide^23^ should make data reporting a priority on the same scale as design and analysis.

Future scoping reviews would be useful for comparing the transparency across the various technical subtypes (e.g. in vitro, in vivo) or disciplines (e.g. neuroscience, immunology, developmental science) in preclinical science. In particular, the inclusion of non-animal (cell) research in the present work is distinctive since reporting is often not presented as a component of reproducibility in research conducted in vitro^24^. Future investigation on the quality of reporting in research made in vitro might be useful. Similarly, the results obtained in the present study cannot be extrapolated to fields other than preclinical science due to cultural differences in data handling and biostatistics across disciplines. Comparable studies in other biological fields of life sciences might therefore provide a broader perspective on statistical reporting in life science. It should also be noted that the transparency in reporting is not an indicator of the quality and appropriateness of the statistical analysis performed.

The present study has limitations. First, other statistical items linked to data presentation could have been included such as the unambiguous reporting of errors or unsound choices of graphical display^18, 25^. Furthermore, the sample used might not be fully representative of the entire population of preclinical publications due to a relatively small sample size or a possible end-of-year bias. The relatively small sample used (n = 16 journals) might also have precluded to reach sufficient statistical power in the corelation study (Fig. 2). The sample also contains a relative over-representation of some editors, which might give some bias. In addition, the scoping review has been designed and performed by one single reviewer, a protocol that might increase error and bias^26, 27^.

In conclusion, this work provides a rigorous documentation of sub-optimal statistical reporting in the specific field of preclinical sciences. It prompts more active enforcement of existing guidelines or the creation of unified recommendations. The systematic inclusion of data presentation, in addition to design and analysis, in undergraduate or postgraduate statistical education is strongly encouraged.

## Methods

### Data collection, statistical analysis and presentation

Data were collected, organised and processed using Microsoft Excel for Mac (version 16). GraphPad Prism for Mac (version 8, GraphPad Software LLC) was used to calculate medians, interquartile ranges (IQR), Spearman’s rank order correlations and to create graphs. Non-parametric Spearman correlation was chosen during the study design due to the anticipated existence of a marked skew of the distribution of journal impact factor. For quantitative features (number of figures and tables) and binary items (i.e. measuring the number of elements incompletely reported, Figs. 1 and 2), journals were used as observational units and articles were sampling units due to the possible confounding influence of journal policies. For qualitative items (Fig. 3), results were aggregated for the whole dataset, each article being both an observational unit and a sampling unit (k = 223). Sample sizes (observational units) are shown in figures and figure legends. The manuscript was prepared following the PRISMA-ScR extension of the PRISMA guidelines for scoping reviews^28^. This study was not preregistered.

### Article sampling

**Figure 4:**
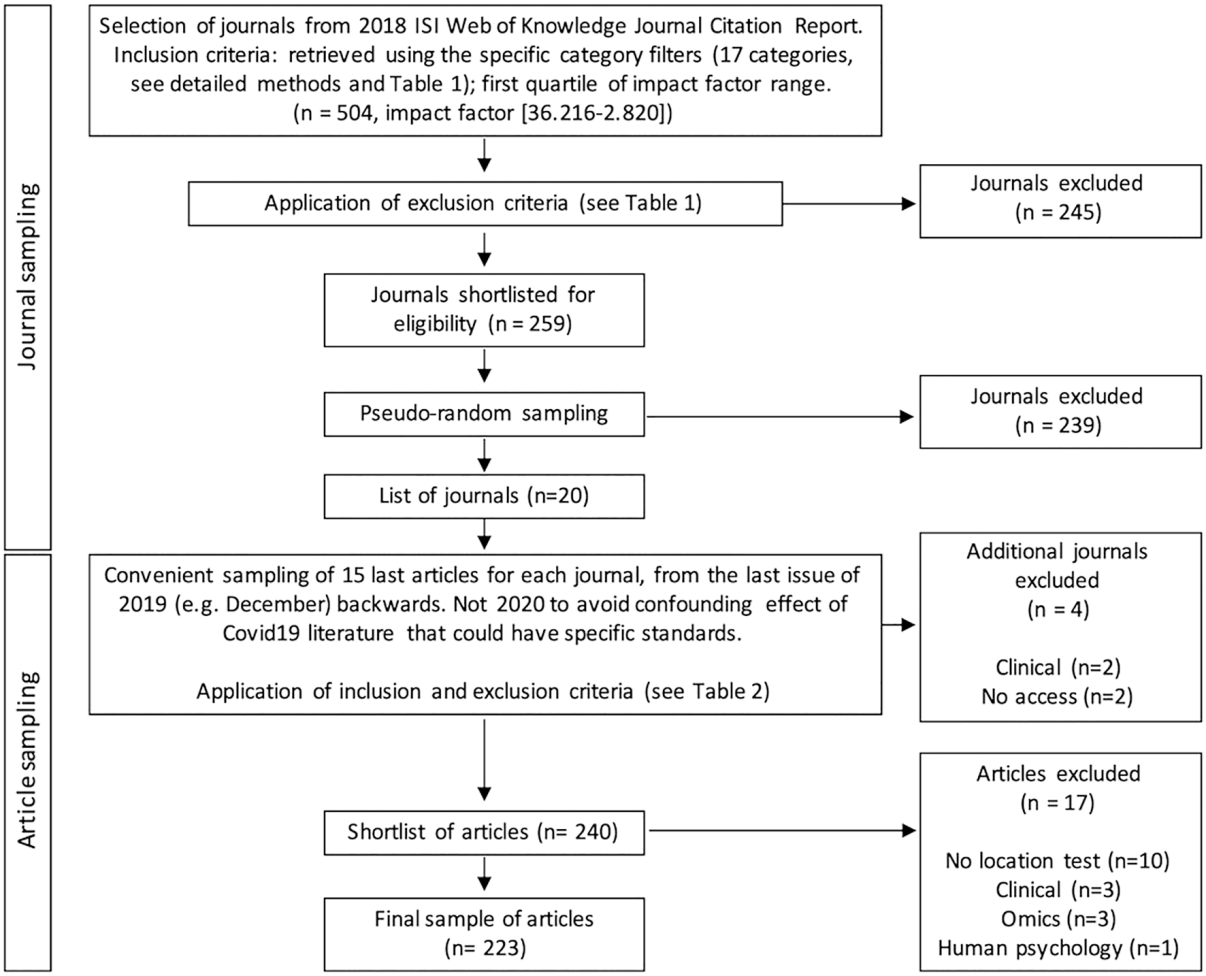
Flow chart of the sampling protocol.

A mixed sampling methodology was implemented (Fig. 4) to collect journals and articles. First, a selection filter was applied within the Institute for Scientific Information (ISI) Journal Citation Report (https://jcr.clarivate.com) database to generate a list of 504 life science journals. Then, exclusion criteria were applied to the journal list and 245 periodicals were removed. Filters and exclusion criteria are given in Table 1. Using a pseudo-random sequence of 20 numbers between 1 and 259 generated using GraphPad QuickCalc (https://www.graphpad.com/quickcalcs/randMenu), a final shortlist of 20 journals among the 259 preselected ordered by decreasing 2018 Impact Factor were selected (the latest available impact factor at the time of designing this study). Four additional journals were finally excluded either because they were eventually found to be too clinical or because there was no online access granted to the author’s institution, leading to a final list of 16 periodicals (Table 2). Clinical journals were excluded although they may include publications with some preclinical experiments. This was justified to prevent the possible bias created by both the presumed small proportion of such articles in clinical periodicals which would have prompted a larger sampling and the supposed compliance of these studies with clinical guidelines whose standards may be different^29, 30^.

**Table 1:** Selection criteria applied to the ISI JCR database to get the first list of 504 life science journals and exclusion criteria applied subsequently to obtain the final list.

Year	2018
Impact Factor quartile	Q1
Categories (in alphabetical order)	Behavioral sciences; Biomedical research methods; Biochemistry & Molecular biology; Biology; Cell &; Tissue engineering; Cell biology; Developmental biology; Genetics & Heredity; Immunology; Microbiology; Multidisciplinary sciences; Neurosciences; Oncology; Pharmacology & Pharmacy; Physiology; Psychology; Biological; Psychology; Experimental; Toxicology; Virology
Exclusion criteria	Reviews only; Clinical only; Human psychology only; Veterinary; Zoology; Ecology; Computational; Biotechnology only; Structural biology only; Non-English language; Duplications

**Table 2:** Final list of journals included in the study (k = 16).

Journal name	2018 IF	Exclusions	Final sample
Science	41.06	3	12
Cancer Cell	23.92	3	12
Nature Neuroscience	21.13	2	13
Science Translational Medicine	17.20	0	15
Journal of Pineal Research	15.22	0	15
Biological Psychiatry	11.50	0	15
Cancer Immunology Research	8.62	1	14
Cell Death and Differentiation	8.09	1	14
Neuropsychopharmacology	7.16	1	14
Science Signaling	6.56	1	14
Molecular Therapy—Oncolytics	5.71	1	14
Cellular Oncology	5.02	0	15
European Journal of Pharmaceutics and Biopharmaceutics	4.71	0	15
Neuropharmacology	4.37	0	15
Journal of Virology	4.32	1	14
Toxins	3.89	3	12

Fifteen articles per journal were collected by sampling the online contents of each journal, starting from the last issue released in 2019 and browsing backward. This time window was selected to avoid the abundant literature on Coronavirus disease 2019 (Covid-19) published since January 2020, which might show unusual statistical standards. Article inclusion and exclusion criteria are presented in Table 3. Studies using human data were acceptable when they used ex-vivo/in-vitro approaches for extracting tissues, cells or samples. From this intermediate list of 240 articles, 17 were finally excluded during the analysis due to previously unnoticed violations of inclusion criteria or for congruity with exclusion criteria, resulting in a final sample set that included 223 articles.

**Table 3:** Inclusion and exclusion criteria applied to articles.

Inclusion criteria	At least one experimental design in biology or human-related pathology (i.e. preclinical animal model, in-vitro, ex-vivo); at least one design with replication (i.e. multiple experimental units); use of location test in at least one figure/table
Exclusion criteria	Review articles; only clinical; case reports; only human psychology; meta-analysis; veterinary; zoology; ecology; computational; biotechnology-only; only structural biology; unusual journal issue (e.g. supplement, meeting papers); absence of location test

### Assessment of reporting

Each article was explored, and three types of statistical attributes were quantified (Table 4). Indicators of the transparency of study protocols were binary items coded as 0 (presence of all needed information in the text) or 1 (absence of information in the text for at least one figure or table) and were aggregated as proportions of articles that had an insufficiency (non-disclosure) for the given item. The indicators were chosen as the minimum set of information needed by a reader to replicate the statistical protocol: precise sample size (experimental units), well identified test, software and no contradiction. The article structure was assessed using quantitative items, specified as total counts of given items as well as one binary outcome (presence of a statistical paragraph). Qualitative items represented the article content and have been summarised as an inventory of information of interest. In the sampled articles, supplemental methods and information were considered full-fledged methodological information, but supplementary figures and tables presenting results were not eligible for the quantification of statistical insufficiencies, even if they were used to report location tests.

**Table 4:** Description of items used to quantify the insufficiencies in reporting in articles.

Type of items	Description
Binary	Presence of a dedicated statistical paragraph Unambiguous disclosure all statistical tests performed Disclosure of statistical software used Unambiguous disclosure of all exact sample sizes Absence of contradictory information about methods
Quantitative	Total number of figures and tables Number of figures with location tests
Qualitative	List of all statistical location tests/procedures used List of all statistical software/packages used

## Data Availability

The dataset generated during this study is available in the Figshare repository (https://doi.org/10.6084/m9.figshare.13385621).
